# New Insights into Cardiac Ion Channel Regulation 2.0

**DOI:** 10.3390/ijms24054999

**Published:** 2023-03-05

**Authors:** Brian P. Delisle, Ademuyiwa S. Aromolaran

**Affiliations:** 1 Department of Physiology, 741 S Limestone Street BBSRB B353, Lexington, KY 40536, USA; 2 Department of Surgery, Division of Cardiothoracic Surgery, Nora Eccles Harrison Cardiovascular Research and Training Institute, Salt Lake City, UT 84112, USA; 3 Department of Biomedical Engineering, Nutrition and Integrative Physiology, Biochemistry and Molecular Medicine Program, University of Utah School of Medicine, Salt Lake City, UT 84112, USA

Sudden cardiac death (SCD) and arrhythmias represent a global public health problem, accounting for 15–20% of all deaths [1,2]. The past 30 years have seen improvements in cardiovascular (CV) care that have decreased the risk for SCD, but there is still much to be learned about the numerous and complex mechanisms that cause SCD. Evidence from both clinical and animal studies have shown that the pathological remodeling of major cardiac ionic channels contributes prominently to arrhythmias/SCD and further highlights a key role for ion channel biophysical properties and the normal sinus rhythm. The Special Issue *New Insights into Cardiac Ion Channel Regulation 2.0* presents a series of articles that provide new and important mechanistic insights into ion channel biophysics, which are expected to help guide future studies that continue to decrease the risk for SCD.

The Special Issue opens with a research article by Chowdhury and colleagues which is based on the premise that dietary obesity elevates the risk for CV disease, particularly the incidence for life-threatening arrhythmias that increases propensity for SCD in patients. Although it is known that obesity and its associated pathologies (including diabetes and insulin resistance) contribute to the prevalence of ventricular arrhythmias, more work is required to understand the mechanism(s) by which obesity elevates the risk for CV disease. One reason more work is needed is because the pathophysiology of obesity is complex, involving individual and multiple combinations of pathological cellular remodeling that ultimately provides triggers and substrates for the initiation and maintenance of arrhythmias. In obesity, the heart responds to metabolic stress through the marked accumulation of adipose tissue, leading to cardiac lipotoxicity (or the abnormal accumulation of free fatty acids in the heart) [3], which likely plays a critical role in the pathological remodeling of ventricular electrical activity, leading to delayed repolarization and the prolongation of heart-rate-corrected QT intervals, and also predisposes patients to life-threatening polymorphic ventricular tachycardia such as torsades de pointes. Thus, understanding the cellular proarrhythmic mechanisms of lipotoxicity is likely to provide novel and additional insights into obesity-related arrhythmias.

Chowdhury and colleagues [4] tested the hypothesis that obesity-related lipotoxicity induced profound adverse ventricular electrical activity via chronic elevations of proinflammatory cytokines. In the study, using a combination of approaches (including electrophysiology, biochemical, and in silico), the authors revealed that the proinflammatory cytokine, interleukin (IL)-6, promotes arrhythmogenesis and severely blunts the densities of the rapid (*I_Kr_*) and slow (*I_Ks_*) components of the delayed rectifier K current, and therefore may contribute to lipotoxicity-induced QT_c_ prolongation and vulnerability to life-threatening arrhythmias. Importantly, these findings suggest the intriguing potential for anti-IL-6 trans-signaling therapy for the prevention of life-threatening ventricular arrhythmias in obese patients. The potential of the IL-6 trans-signaling pathway as a promising therapeutic target has also been demonstrated in a Duchenne muscular dystrophy mouse model [5]. The clinical significance of IL-6 trans-signaling therapy is further highlighted by the Canakinumab Anti-Inflammatory Thrombosis Outcomes Study (CANTOS). In this trial, Ridker et al. [6] demonstrated that although canakinumab, a human therapeutic monoclonal antibody targeting IL-1β, significantly reduces major adverse cardiovascular events, participants remained at an increased risk for recurring cardiovascular events, particularly those with the highest pathological levels of IL-6. Thus, specific IL-6 trans-signaling modulators have the potential to enhance the efficacy and even exceed the beneficial effects of anti-inflammatory drugs that are currently used in clinical trials. Further studies addressing the molecular and functional basis of cytokine-related arrhythmogenesis are starting to emerge and further research is warranted.

The upstroke or initial phase of the cardiac action potential is controlled by the influx of Na currents (*I_Na_*) through voltage-gated Na channels, specifically Na_v_1.5, which is encoded by the *SCN5A* gene. Another research article [7] by Marcos Rubio-Alarcón and colleagues builds on their earlier work on the transcriptional regulation of major atrial ion channels [8], and provides evidence for how the zinc finger homeobox 3 (Zfhx3) transcription factor may regulate the functional expression of Na_V_1.5 channels. The authors provide compelling evidence for the multiple effects of Zfhx3 on the functional expression of *SCN5A* that leads to a severe depression of peak *I_Na_* density. These effects include acting as a repressor on the *SCN5A* promoter, preventing the Tbx5-dependent facilitation of *I_Na_* density, and increasing the expression of Nedd4-2. This elegant study highlights the complex relationships between ion channel dysfunction, pathological cardiac electrical excitability, and the increased risk for arrhythmias (particularly atrial fibrillation). Additionally, Daimi and colleagues [9] provide a timely review of the molecular events that underlie the functional regulation of the *SCN5A*/Na_V_1.5 channel, and discuss how channel protein dysfunction may cause cardiac channelopathies and promote associated arrhythmias. The modulation of Na_V_1.5 channel gating by the neurotoxin veratridine (VTD) is the focus of the research article by Gulsevin and colleagues [10]. An important implication of their findings is that VTD binds to a site close to the cytoplasmic mouth of the channel pore, which may therefore play an important role in the VTD-dependent allosteric inactivation of the Na_V_1.5 channel. Together, these articles highlight the pathophysiology of *SCN5A*/Na_V_1.5 and identify new opportunities in the development of novel anti-arrhythmia therapies.

The research article by Wei Hu and colleagues [11] is a comprehensive and rigorous electrophysiological and computational evaluation of the biophysical properties (including gating kinetics and sensitivity to ionic and organic inhibitors) of the hyperpolarization-activated nonselective cation current (*I_f_*). The study reveals novel biophysical insights into the regulation of the *I_f_* channel, which are likely to be informative in the development of efficient and clinically relevant approaches that regulate cardiac automaticity.

Finally, the article by Aromolaran and colleagues describes a novel mechanism involving the mTOR complex 1 (mTORC1, protein translation pathway) modulation of atrial myocyte electrical activity [12]. The authors demonstrate a potential role for the overactivation of mTORC1 activity in the progression of atrial arrhythmogenesis (shortened action potential duration, increased *I_Kr_* current density, and gating defects), due to lipotoxicity or high-fat diet feeding. An important demonstration from this study is that this occurred through a targeted effect on hERG1b protein translation, but was independent of on-going transcription. In light of the findings of Marcos Rubio-Alarcón and colleagues, and a recent study [13] from Dr. Gail Robertson’s laboratory that elegantly presented evidence for the co-translational association and regulation of Na_V_1.5 and hERG transcripts, it is tempting to speculate about the cellular mediators that prevent the transcription and/or translation of *SCN5A*/Na_V_1.5 protein channel subunits, or promote hERG1b protein expression, which may limit pathological atrial arrhythmogenesis and decrease the risk for supraventricular arrhythmias. Whether and how such reciprocal regulation of Na_V_1.5/hERG channel protein subunits may occur in metabolic obesity, a prominent contributor to the prevalence of arrhythmias warrants further investigation.

In summary, the articles published in the Special Issue *New Insights into Cardiac Ion Channel Regulation 2.0* continue the discussion on, and provide new information about, the complex modulation of cardiac ion channels that impact the electrical activity of the heart. Advancing research that explores inherited and acquired channelopathies will advance the arrhythmia field in ways that provide new opportunities for the development of novel therapies that reduce the risk for SCD and improve the quality of life for people living with heart disease.

## Data Availability

All the relevant data are included within the paper itself.
